# Bipolar Switching Properties and Reaction Decay Effect of BST Ferroelectric Thin Films for Applications in Resistance Random Access Memory Devices

**DOI:** 10.3390/nano15080602

**Published:** 2025-04-14

**Authors:** Yao-Chin Wang, Kai-Huang Chen, Ming-Cheng Kao, Hsin-Chin Chen, Chien-Min Cheng, Hong-Xiang Huang, Kai-Chi Huang

**Affiliations:** 1 Department of Electronic Engineering, Cheng Shiu University, Kaohsiung 83347, Taiwan; 0644@gcloud.csu.edu.tw (Y.-C.W.); 0662@gcloud.csu.edu.tw (H.-C.C.); m1103105@gcloud.csu.edu.tw (H.-X.H.); 2 Graduate Institute of Aeronautics, Department of Information and Communication Engineering, Chaoyang University of Technology, Taichung 413310, Taiwan; 3 Department of Electronic Engineering, Southern Taiwan University of Science and Technology, Tainan 71005, Taiwan; ccmin523@gmail.com (C.-M.C.); chiashu.lin@gmail.com (K.-C.H.)

**Keywords:** RRAM, BST, bipolar switching properties, thin film, sputtering

## Abstract

In this manuscript, strontium barium titanate (BST) ferroelectric memory film materials for applications in the feasibility of applying to non-volatile RAM devices were obtained and compared. Solutions were synthesized with a proportional ratio and through the deposition of BST films on titanium nitride/silicon substrates using the sol–gel method, using rapid thermal annealing for defect repair and re-crystallization processing. The crystallization structure and surface morphology of annealed and as-deposited BST films were obtained by XPS, XRD, and SEM measurements. Additionally, the ferroelectric and resistive switching properties for the memory window, the maximum capacitance, and the leakage current were examined for Al/BST/TiN and Cu/BST/TiN structure memory devices. In addition, the first-order reaction equation of the decay reaction behavior for the BST film RRAM devices in the reset state revealed that $r=0.19{\left[O^{2-}\right]}^{1}$. Finally, the Cu/BST/TiN and Al/BST/TiN structures of the ferroelectric BST films RRAM devices exhibited good memory window properties, bipolar switching properties, and non-volatile properties for applications in non-volatile memory devices.

## 1. Introduction

With the rapid development of technology in recent years, electronic products have been continuously and rapidly changing. Various semiconductor technologies have been continuously evolving, making electronic products lighter, shorter, and smaller with enhanced functions and efficiency. As these memory devices functions continuously develop, the demands of memory devices demand have also increased. Functional thin films are primarily used in non-volatile RAM (NvRAM) applications, such as electronic cards and 3C devices, because of their good memory characteristics, capacity, retention, power consumption, non-volatility, and readout properties. Recently, resistive memory has gained attention in artificial intelligence and synapse-like components for its quick switching speed and low energy usage [1,2,3,4].

Recently, the various non-volatile RAM devices such as ferroelectric RAM (FeRAM), magnetoresistive RAM (MRAM), resistive RAM (RRAM), and flash memory were extensively studied and discussed [1,2,3,4,5,6]. However, the volatile chemical pollution elements and high processing costs associated with complex composite materials exhibit significant challenges for their application in integrated circuit semiconductor processing. Consequently, binary metal oxide materials such as Al_2_O_3_, ZnO, Ta_2_O_5_, and TiO_2_ have been widely discussed and researched for applications in different functional electronic products, especially in resistive RAM devices [7,8,9].

Resistive RAM (RRAM) devices currently utilize various materials for switching films in top metal–insulator–bottom metal (MIM) structures, as well as for HfO_X_, TiO_X_, NiO_X_, GdO_X_, TaO_X_, and ABO_3_ perovskite structures [10,11,12,13,14,15,16,17,18,19,20,21,22,23]. The forming process’ working principle for the RRAM devices involves applying a positive and negative bias to one electrode to form the conductive mechanism within the switching films, which creates the conduction transport path with the electrode. Based on the formation method of the conductive path, RRAM devices might be categorized as oxide-based memory devices (OxRAM) and conductive-bridge RAM (CBRAM) devices. For OxRAM devices, the conductive path is formed by oxygen vacancies, allowing current to pass through. When applying positive and negative bias to the device, the conductive path forms or breaks, switching the device between LRS and HRS states to store data. For CBRAM devices, the conductive path relies on active electrodes, composed of, for example, Cu and Ag. The path is formed by the reaction and migration of metal ions [24,25]. CBRAM devices are notable for their high switching ratio, low operating voltage, and compatibility for semiconductor fabrication processes.

Previous studies have identified ABO_3_ perovskite and Bi-layer ferroelectric (BLSF) materials as promising materials for various ferroelectric RAM (FeRAM) device applications. The large remnant polarization and good coercive field properties of materials like (Pb(Zr,Ti)O_3_, PZT), (Sr_2_Bi_2_Ta_2_O_9_, BST), (Ba(Zr,Ti)O_3_, BZT), and ((Ba,Sr)TiO_3_, BST) in the ABO_3_ structure have been extensively studied to realize the good storage capacity of FeRAM devices. BST and BZT ferroelectric materials have been considered potential substitutes for PZT or BST materials to mitigate environmental pollution because of their low ecological impact. Additionally, the dielectric constant and leakage current density properties of BST thin films make them suitable for various electronic device applications [26,27,28,29,30,31].

Recently, numerous studies have focused on the application of ferroelectric thin film materials such as ABO_3_ perovskite and Bi-layer ferroelectrics (BLSFs) in varistor memory device technology using various oxide materials [26,27,28]. This study will examine the feasibility of utilizing BST ferroelectric thin film materials in the construction of RRAM devices for oxide-based memory and conductive-bridge random access memory devices. Additionally, it will discuss the bipolar switching properties and reaction decay effects according to first-order reaction law in BST ferroelectric thin films for applications in resistance random access memory devices.

## 2. Experiment

This study used the sol–gel method to synthesize a barium strontium titanate (BST) solution, which was then deposited onto a TiN/Si base using spin coating to form a switching layer for non-volatile RRAM device applications. The BST precursor solution was prepared by dissolving barium acetate [Ba(CH_3_COO)_2_], strontium acetate [Sr(CH_3_COO)_2_], and titanium isopropoxide [Ti(OCH(CH_3_)_2_)_4_] in a mixed solvent of glacial acetic acid and ethylene glycol. The molar ratio of Ba:Sr:Ti was fixed at **0.6:0.4:1**, with the total metal concentration adjusted to **0.5 M**. The solution was stirred magnetically at 60 °C for 1 h to ensure homogeneity, then concentrated using a rotary evaporator at 70 °C to reduce solvent content, and aged at room temperature for 24 h. The BST film was deposited on the TiN substrate at 1000 rpm for 10 s and then 4000 rpm for 30 s, and then heated at 250 °C for 10 min to remove organic solvents. For the pre-deposition process at 25 °C, the as-deposited BST films was prepared using rapid temperature annealing (RTA). In addition, the as-deposited BST films underwent a 30 s post-treatment process, at annealing temperatures of 400 °C, 500 °C, and 600 °C to crystallize and repair the BST films. The minimum temperature that initiates the formation of the perovskite phase is400 °C, and higher annealing temperatures (500 and 600 °C) are expected to reduce defects and improve film quality.

**Figure 1:**
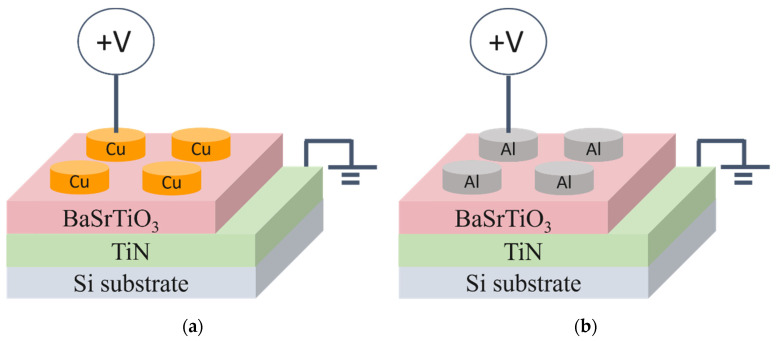
The micro-structure of the BST film RRAM devices for (**a**) Cu/BST/TiN/Si and (**b**) Al/BST/TiN/Si structures.

The MIM structure devices, prepared with Cu and Al as top electrodes, were examined for the bipolar switching characteristics of the Cu/BST/TiN and Al/BST/TiN structure RRAM devices in Figure 1. These were created using dc sputtering, with a sputtering power of 200 W for TiN material, followed by Al top-electrode material deposition via thermal evaporation. The micro-structure of the BST films was obtained using field emission scanning electron microscopy (Philips Tecnai G2 F20 FEG-STEM, FE-SEM, OR, USA). Additionally, the preferred phase of the BST films was determined through x-ray photoelectron spectroscopy (JEOL, JAMP-9500F, XPS, Tokyo, Japan) and *x*-ray diffractometer (Bruker D2 PHASER, XRD, Germany) measurements. The *I*-*V* switching characteristics of BST film RRAM devices were analyzed with an Agilent B1500 semiconductor parameter analyzer (CA, USA). Measurements of the *p*-*E* hysteresis loops (RT66A (Radiant-tech, Lewis Center, OH, USA) were conducted using a Sawyer-Tower circuit in the MIM structures of Cu/BST/TiN and Al/BST/TiN RRAM devices.

## 3. Results and Discussion

**Figure 2:**
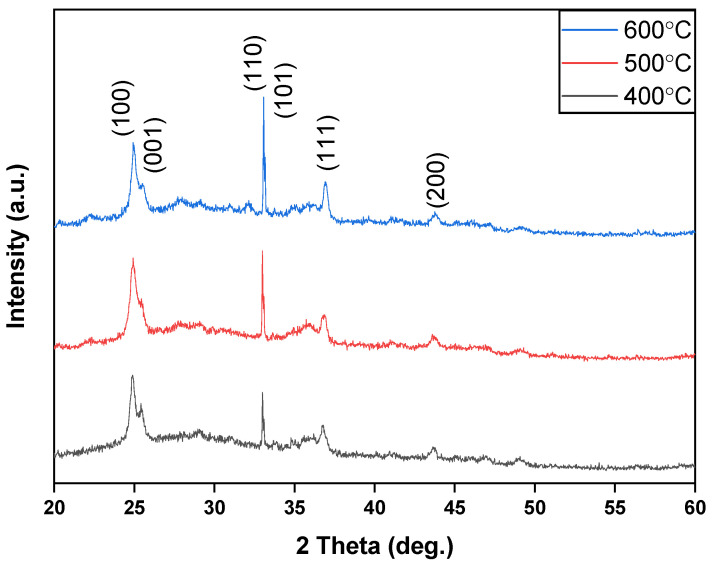
XRD patterns of the BST film RRAM devices for different annealing temperatures.

Figure 2 shows the XRD measurement results of BST films at different annealing temperatures. In Figure 2, it is observed that the main preferred crystal phases of the BST film were at (100), (101), and (110), with the preferred peak diffraction angles approximately around 25, 32, and 37 degrees, respectively. These peaks align well with the standard diffraction data of BST (JCPDS No. 34-0411), confirming the formation of a crystalline perovskite structure. According to Bragg’s Law, for the (100) peak of 2θ = 24.9° to 25°, the lattice constants range from 3.573 to 3.55 Å; (110) peak of 2θ = 33° to 33.08°, 3.835 to 3.826 Å; and (111) peak of 2θ = 36.7° to 33.9°, 4.237 to 4.215 Å, as shown in Figure 2. It also looks like the peaks shifted to high angles at higher temperatures, which indicates that the lattice constants decrease. Additionally, the smallest full-width at half-maximum (FWHM) value for the (101) and (100) preferred peaks of the BST films at various annealing temperatures was recorded. The BST films annealed at 600 °C demonstrated the smallest FWHM value compared to other temperature treatments. The grain sizes were calculated, resulting in a size of 0.299 µm for the 400 °C annealing, 0.282 µm for 500 °C, and 0.352 µm for 600 °C.

**Figure 3:**
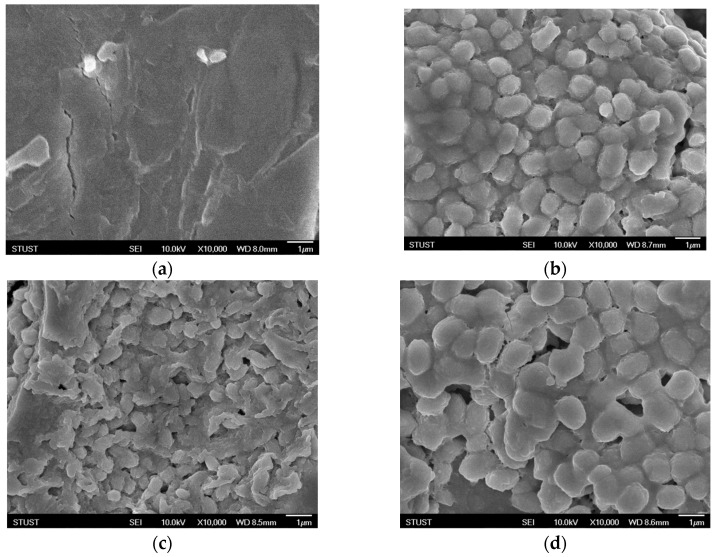
The micro-structure of the BST film RRAM devices for the different annealing temperatures: (**a**) as-deposited, (**b**) 400 °C, (**c**) 500 °C, and (**d**) 600 °C.

SEM images of the surface micro-structure of the ferroelectric strontium barium titanate oxide film of the RRAM devices are shown in Figure 3. The specimens were approximately about 30–50 nm for the 500 °C annealing temperature treatment, exhibiting uniform grains. Normal oval-style grain structures are shown in Figure 3. Figure 3a presents a lack of significant grain growth on the surface of the non-treated BST films. For an annealing temperature of 400 °C, grain growth on the film surface is apparent, but large damage defects were also obtained. The grain size of BST films was approximately 0.3 µm. At an annealing temperature of 500 °C, the holes on the surface of the BST film were repaired, improving the defects, as shown in Figure 3c. However, for the annealing temperature of 600 °C, the crystal grains melted off the BST films, as shown in Figure 3d. Additionally, the grain sizes for films annealed at 500 °C and 600 °C were about 0.282 µm and 0.352 µm, respectively. In our previous study, the crystallization quality, the dielectric loss, and leakage current properties of as-deposited films showed improvement for films exposed to various oxygen annealing treatments. Additionally, the annealing process reduced the number of traps and vacancies in the films, leading to the lowering of the leakage current density.

**Figure 4:**
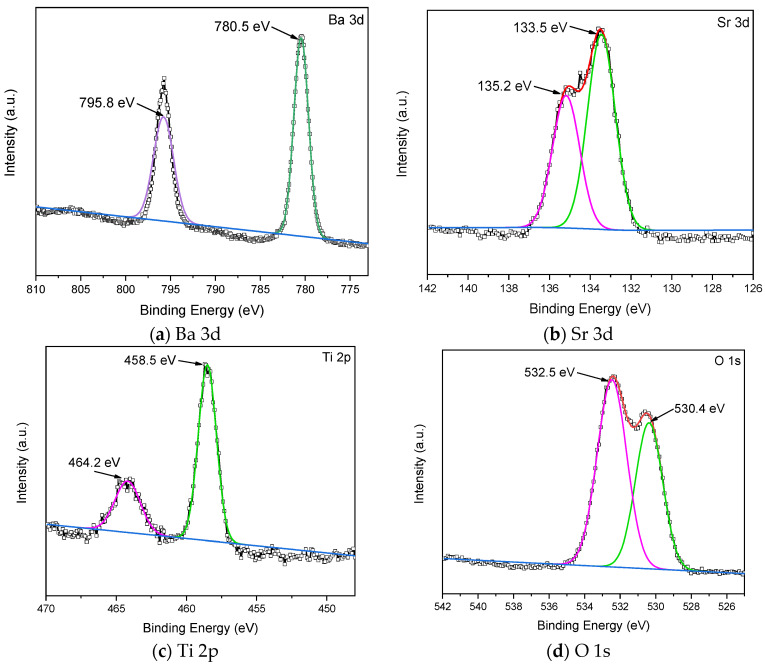
XPS results of the BST films for (**a**) Ba 3d (**b**) Sr 3d (**c**) Ti 2p, and (**d**) O 1s peaks.

X-ray photoelectron spectroscopy (XPS) was carried out on barium strontium titanate films, as shown in Figure 4. The surface analysis of the film annealed at 500 °C identified barium, strontium, titanium, and oxygen. Figure 4 presents the surface composition analysis obtained through XPS measurements in a range of 0–1300 eV for BST films, for the peaks of (a) Ba 3d, (b) Sr 3d, (c) Ti 2p, and (d) O 1s. The binding energy for Ba 3d in the BST films is identified at 780.5 eV and 795.8 eV in Figure 3a. Figure 4b presents the energy spectrum of the Sr element, for the peaks at 133.5 eV and 135.2 eV. Figure 4c shows the energy spectrum for the Ti element, with peaks at 458.5 eV and 464.2 eV. Figure 4d shows the energy spectrum for the O element, indicating peaks at 530.4 eV and 532.5 eV. The results show the following: Ba: 6.62%; Sr: 7.92%; Ti: 6.79%; and O: 78.67%. The mole fraction ratios of the Ba, Sr, Ti, and O elements in the RTA-treated BST films were obtained from the XPS measurement results.

**Figure 5:**
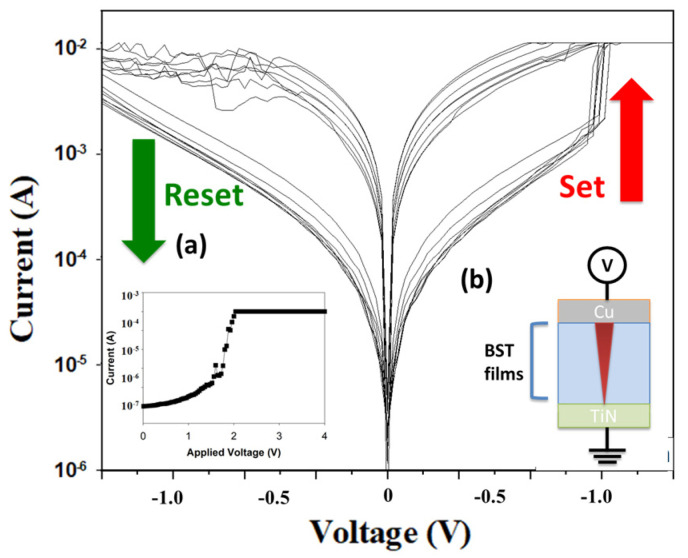
The *I*-*V* curves of the BST film RRAM device using a copper top electrode for the 500 °C annealing temperature for (**a**) initial forming process, and (**b**) Cu/BST/TiN structure.

Figure 5a shows the properties of BST film RRAM devices for the initial forming process in set states. In addition, the Al(Cu)/BST film/TiN RRAM device structure is also shown in Figure 5b. For the initial forming process, applying a bias during the forming step creates the conductive path inside the device, resulting in the soft breakdown that switches the state from HRS to LRS. Subsequently, the positive and negative biases were applied to the RRAM device to perform back-and-forth sweeping, allowing the resistance state to toggle, thus determining the set and reset states. As seen in Figure 5, a current compliance of 10 mA was used, and the initial forming process occurred at about 2 V when using Cu as the top-electrode material. When using aluminum as the top-electrode material, the threshold voltage was about 1 V for the initial formation process. To achieve stable *I*-*V* curves, the BST film RRAM device was repeatedly set to LRS by applying a high positive bias above the set voltage and reset to HRS with the negative bias over the reset voltage. This process was repeated 100 times in Figure 5.

**Figure 6:**
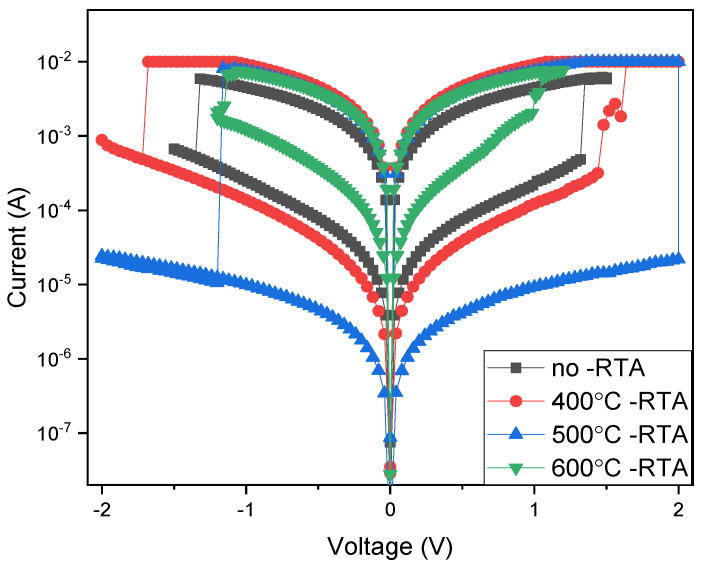
The *I*-*V* curves of the BST film RRAM device using an Al top electrode for the different annealing temperatures.

Figure 6 presents the current–voltage (*I*-*V*) property curves of the BST film RRAM devices using the aluminum top-electrode material. As illustrated in Figure 6, the on/off ratio was approximately 1 for as-deposited BST films. With an annealing temperature of 400 °C, the on/off ratio increased to about 3. As the annealing temperature reached 500 °C, the increased memory window of the BST switching layer was repaired and internal defects decreased. Consequently, the memory windows’ on/off ratio increased; this might be attributed to the internal grains and defect reduction of the BST switching layer in RRAM devices for the temperature of 500 °C. For the temperature of 600 °C, the on/off ratio of the film RRAM devices decreased to approximately 0.5. According to the measurement results, the BST films crystallized, and the memory window characteristics of the film RRAM devices improved due to the following different annealing temperature processes. In addition, the electrical properties of the film RRAM devices were caused by the excessively high annealing temperatures inducing defects in the BST films. Therefore, the optimal annealing temperature of 500 °C was determined to be a preferred fabrication processing parameter for BST films utilizing the aluminum top electrode for film RRAM devices.

**Figure 7:**
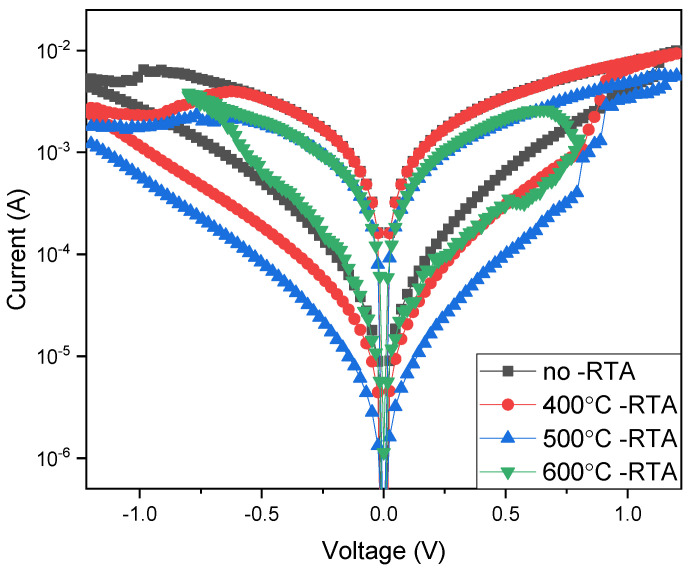
The *I*-*V* curves of the BST film RRAM device using a copper top electrode for the different annealing temperatures.

Figure 7 presents the *I*-*V* curves of RRAM devices for using copper top-electrode materials. As depicted in Figure 7, the on/off ratio was approximately 1.2 for the annealing treatment ranging 400–600 °C. The on/off ratio was increased to about 1.5 for an annealing temperature of 400 °C, as shown in Figure 7. At the annealing temperature of 500 °C, the internal defects in the BST switching layer were repaired. This resulted in an increased memory window and the memory window’s on/off ratio of 2. In addition, the high annealing temperature of 600 °C might have caused the internal grains of the BST switching layer to melt. This also resulted in defects that decreased the memory window’s characteristics. The memory window’s on/off ratio was approximately 1. From the measurement results summarized and depicted in Figure 7, it is evident that the crystallization of the BST films was repaired as the annealing temperature increased. In addition to the memory window characteristics of film RRAM devices, annealing temperature increases were also improved. When using the copper top electrode, the optimal annealing temperature of 500 °C was also determined to be the preferred fabrication processing parameter for BST film RRAM devices. Finally, the on/off ratio drops at 600 °C for many reasons, such as excessive grain growth and the interfacial reactions of BST films for the *I*-*V* curves of the RRAM device when using copper and aluminum top electrodes for the different annealing temperatures. This was verified in the following electrical conduction mechanism by conducting a simulation analysis of the metallic filament conduction behavior.

To examine the electrical conduction mechanism of the metallic filament conduction, the ohmic conduction and hopping conduction mechanisms were analyzed using *I*-*V* and ln*I*-*V*^1/2^ curve fitting. The calculation of the *I*-*V* curve involved transforming the ohmic conduction mechanism function based on the *I*-*V* curves of the RRAM devices. Regarding ohmic conduction,

(1) $$J=qunEexp\left[\frac{-\left(\Delta E_{ac}\right)}{kT}\right]$$

where *Eac* is electron activation energy, q is the carrier mobility, *E* is applied electrical field, *n* is the electronic concentration, *k* is the Boltzmann constant, and T is the absolute temperature.

Additionally, to determine the $\ln\left(\frac{I}{T^{2}}\right)-\sqrt{V}$ curve, the hopping conduction behavior function was fitted to the *I*-*V* curves of the RRAM devices. According to the hopping conduction,

(2) $$J=qN_{a}V_{o}e^{-q\Phi_{T}/kT}e^{qaV/2dkT}$$

where Φ*_T_*, *d*, *N_a_*, *a*, and *v*_0_ are the barrier height of hopping, film thickness, density of the space charge, mean hopping distance, and intrinsic vibration frequency, respectively [32,33,34,35,36,37,38,39].

For the fabrication process of the insulating layer of a film RRAM device, when there were defects in the lattice arrangement for the BST films, these defects led to the formation of excess energy levels at the edges of the valence band and conduction band. Consequently, carrier capture by these defects easily occurs in this conduction mechanism. Subsequently, the carriers captured by the defects gain energy to jump the energy barrier and transition into the conduction band or valence band, resulting in an electric field and thermal excitation, as well as hopping and trapping behavior in the insulating layer.

The Poole–Frenkel conduction mechanism is similar to Schottky’s emission behavior. However, it differs in that the mechanism relies on defects capturing carriers, which were thermally excited to enter the conduction band or valence band. Consequently, the energy barrier height in this equation represents the height of the defect potential well, which is given by the following formula [35]:

(3) $$J\propto E\;exp[(-q\left(\Phi_{B}-\surd \left(\left(qE_{i}\right)/\left(\pi \varepsilon_{i}\;\right)\right)/kT\right]$$

where Φ*_B_* is the defect energy barrier height, *E_i_* is the electron field, q is the carrier mobility, *E* is the applied electrical field, *n* is the electronic concentration, *k* is the Boltzmann constant, *T* is absolute temperature, *A* is the Richardson constant, and *ε_i_* is the dielectric constant.

**Figure 8:**
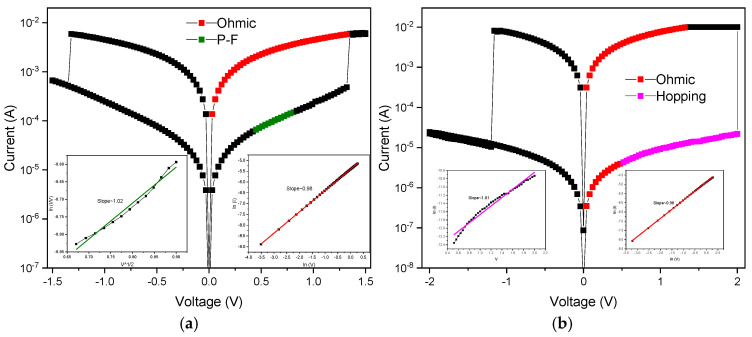
The *I*-*V* curves of the RRAM device using an aluminum top electrode: (**a**) non-treated and (**b**) with 500 °C annealing temperature.

Figure 8a presents a current conduction mechanism distribution diagram for non-annealing processing treatment using an aluminum top electrode in film RRAM devices. According to current conduction mechanism’s fitting results, the mechanism section distribution was known and marked for *I*-*V* curves analysis. The illustration in the low left corner of Figure 8 shows the ohmic conduction mechanism used to convert voltage–current into Ln(*I*)-Ln(*V*) curves for *I*-*V* curve fitting analysis. If the fitting slope is equal to 1, it is consistent with the ohmic conduction mechanism. The illustration in the lower right corner of Figure 8 shows the Poole–Frenkel conduction mechanism used to convert voltage–current into √*V*-Ln (*I/V*) for *I*-*V* curve fitting analysis. If the fitting slope is equal to 1, it complies with Poole–Frenkel conduction. From the *I*-*V* curve analysis results, it was observed that in HRS, electrons were conducted by the Poole–Frenkel conduction mechanism, which means that there were defects in the BST films that cause electrons to fall into the defects and then jump out of them during movement. From the Poole–Frenkel conduction simulation results, it was found that the 600 °C annealing temperature films also showed the mechanism in the HRS state. In addition, this also occurred using the ohmic conduction mechanism for the LRS state.

**Figure 9:**
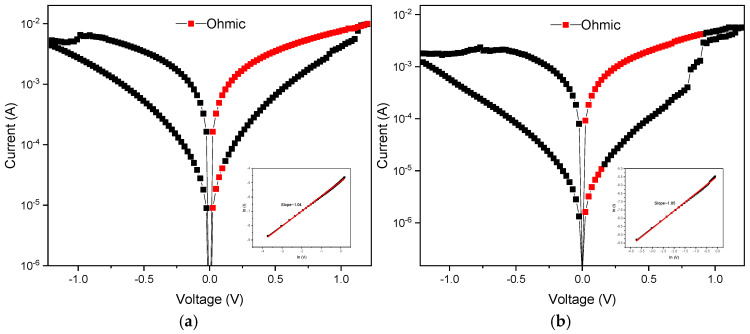
The *I*-*V* curves of the RRAM device using a copper top electrode: (**a**) non-treated and (**b**) with a 500 °C annealing temperature.

Figure 9a presents the current conduction mechanism distribution diagram for non-annealing processing treatment using a copper top electrode in RRAM devices. To fit the results of the current conduction mechanism, the distribution of mechanism sections was indicated and analyzed. Figure 9b presents the *I*-*V* curve fitting analysis by converting voltage–current data into Ln(*I*)-Ln(*V*) using the ohmic conduction formula. If the fitting slope equals 1, it conforms to ohmic conduction. The analysis results indicate that both the HRS and LRS states were dominated by ohmic conduction. Figure 9b shows the distribution diagram of the current conduction mechanism at an annealing temperature of 500 °C. For the fitting results of the current conduction mechanism, the distribution of the mechanism sections was also indicated and analyzed. Figure 9b presents the fitting analysis converting voltage–current data into Ln(*I*)-Ln(*V*) curves using the ohmic conduction formula. If the fitting slope equals 1, it conforms to ohmic conduction. The analysis results indicate that both the HRS and LRS states were dominated by the ohmic conduction mechanism.

**Figure 10:**
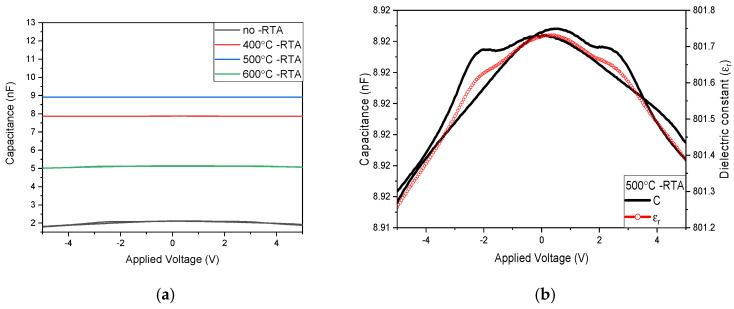
The *C*-*V* curves of the RRAM device using an Al top electrode for (**a**) different annealing temperatures and (**b**) the 500 °C annealing temperature.

As shown in Figure 10a, the capacitance value of the *C*-*V* curves for using non-annealed BST film RRAM devices was approximately 2.12 nF, with a relative dielectric constant of around 189. For annealing temperatures ranging from 400 °C to 600 °C, the capacitance value of film RRAM devices increases to approximately 7.87 nF, and the relative dielectric constant rises to about 707. In Figure 10b, the defects of the insulator layer for the annealing temperature of 500 °C are also repaired, resulting in an increase in capacitance value to 8.92 nF, with a relative dielectric constant of approximately 801. In Figure 10a, the elevated temperature of 600 °C causes the internal grains of the switching layer to melt, leading to an increase in defects and a subsequent decrease in capacitance value. At this point, the capacitance value of film RRAM devices is approximately 5.14 nF, with a relative dielectric constant of around 461. From the compared measurement results in Figure 10b, it is obtained that while increasing the temperature enhances crystallization and repairs the BST thin film, excessively high annealing temperatures may increase defects and worsen their properties. Therefore, the post-treatment temperature of 500 °C was considered optimal.

**Figure 11:**
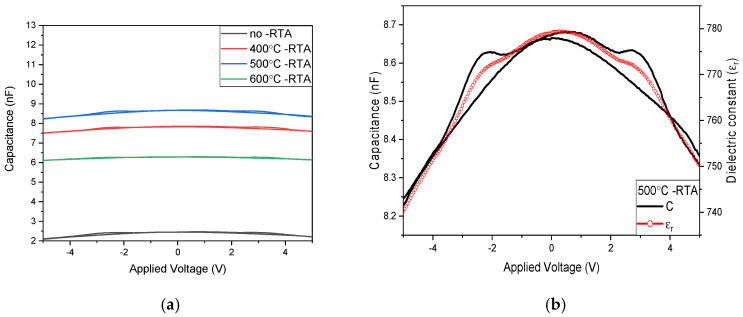
The *C*-*V* curves of the RRAM device using a Cu top electrode for (**a**) different annealing temperatures and (**b**) the 500 °C annealing temperature.

As shown in Figure 11a, the capacitance of as-deposited BST film RRAM devices using a copper top electrode is approximately 2.46 nF, and the relative dielectric constant is about 220 without annealing. For the measurement results after an annealing temperature of 400 °C, the capacitance is about 7.85 nF, and the relative dielectric constant is about 704. Figure 11a presents the results after an annealing temperature of 500 °C: internal defects in the switching layer are repaired, thereby increasing the capacitance to 8.68 nF and the relative dielectric constant to about 779. For an annealing temperature of 600 °C, the excessively high temperature causes the grains in the switching layer to melt, leading to an increase in defects, decrease in capacitance to about 6.29 nF, and decrease in relative dielectric constant to about 565. From the comparison of measurement results in Figure 11b, it is observed that increasing the temperature promotes crystallization and the repair of BST thin films. However, excessively high annealing temperatures may increase defects and degrade the characteristics of the film. Consequently, an annealing temperature of 500 °C for the copper top electrode is considered optimal.

**Figure 12:**
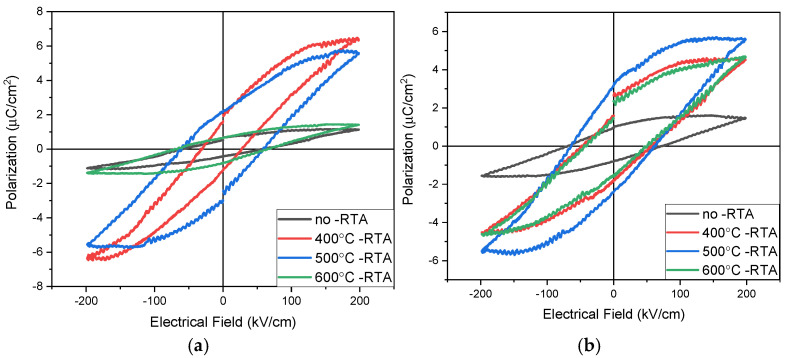
The *p*-*E* curves of the RRAM device using (**a**) aluminum and (**b**) copper top electrodes in the range of 400–600 °C.

Figure 12a illustrates the polarization versus electric field (*p*-*E*) properties for BST film RRAM devices with aluminum top electrodes, measured at 200 kV/cm. The remanent polarization values (Pr) for as-deposited and 400 °C annealed BST films were approximately 0.67 and 1.98 μC/cm^2^, respectively. Increasing the temperature to 500 °C repaired internal defects within the switching layer, thereby enhancing the remanent polarization value. However, with the annealing temperature of 600 °C, the excessively high temperature caused grain melting within the insulating layer, resulting in increased defects and a subsequent decrease in the remanent polarization value. For the 500 °C annealed and 600 °C annealed BST films, the remanent polarization values were approximately 2.2 and 0.7 μC/cm^2^, respectively. Figure 12a demonstrates that increasing the annealing temperature improved crystallization and repaired the films, thereby increasing the remanent polarization value. However, excessively high annealing temperatures introduced more defects, negatively affecting the domain walls of BST films. This decrease in remanent polarization may also be attributed to these elevated temperatures. The importance of the Pr value lies in its ability to maintain spontaneous polarization without an external electric field, indicating that the device can retain internal information without applied voltage, which is crucial for non-volatile memory characteristics.

At 200 kV/cm, Figure 12b shows the relationship of the electric field versus polarization (*p*-*E*) curve properties of BST film RRAM device annealed at different temperatures using copper top electrodes. For the as-deposited and 400 °C temperature BST film RRAM devices, the remanent polarization value Pr was approximately 1.02 and 2.64 μC/cm^2^, respectively. For the 500 °C and 600 °C annealed temperature BST film RRAM devices, the remanent polarization value Pr was approximately 3.29 and 2.34 μC/cm^2^, respectively. In Figure 12b, it is evident that while increasing the annealing temperature can enhance film crystallization and repair, excessively high temperatures can increase defects, affecting domain walls and making polarization reversal difficult, thus reducing the remanent polarization value. The significance of the Pr value is that it maintains spontaneous polarization for external electric field applied. This capability allows the device to store internal information without the need for an applied voltage, thereby fulfilling the requirements for non-volatile memory characteristics. This low remanent polarization value and high coercive electric field reason might be attributed and proved from the interface between the top-electrode materials and copper oxide layer in switching films from the RRAM devices in Figure 12. In addition, the high leakage current might also be verified through the Poole–Frenkel and hopping conduction mechanism from the *I*-*V* curve electrical fitting diagram for the HRS state in Figure 8.

**Figure 13:**
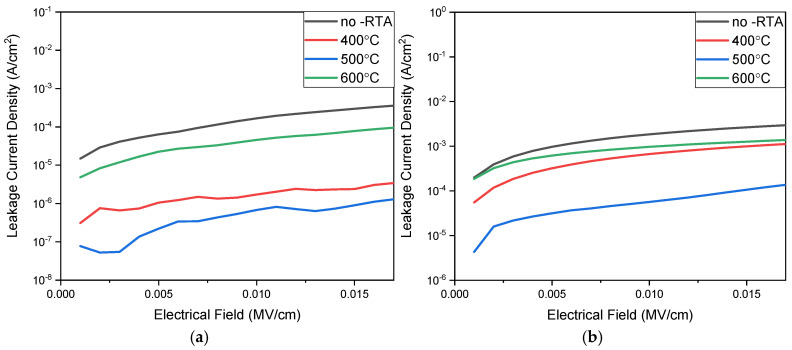
The *J*-*E* curves of the RRAM device using (**a**) aluminum and (**b**) copper top electrodes in the range of 400–600 °C.

Figure 13a illustrates the leakage current measurement results under various annealing temperatures. The *J*-*E* curves of the RRAM device when using an aluminum electrode indicates improved leakage current characteristics after annealing at 400 °C to 500 °C, suggesting that appropriate annealing temperatures effectively eliminate defects within the BST films, release excess stress, and reduce void occurrences. However, excessively high temperatures may cause grain melting and size increases, leading to grain boundary growth and increased BST film leakage current at 600 °C. In addition, Figure 13b illustrates the leakage current measurement results of the RRAM device when using a copper top electrode under various annealing temperatures. The *J*-*E* curves also indicate improved leakage current characteristics after annealing at 400 °C to 500 °C. Figure 13b also indicates improved leakage current characteristics after annealing at 400 °C and 500 °C. In addition, excessively high temperatures might cause grain melting and size increase, leading to grain boundary growth and high increases in film leakage current. Comparing the different top-electrode materials in Figure 13a,b, the *J*-*E* curves of the RRAM device when using the aluminum electrode materials of 10^−7^ A are lower than those using the copper top-electrode materials for 10^−5^ A. This high leakage current might be attributed to and proved by the interface between the top-electrode materials and the copper oxide layer in switching films from the RRAM devices. Similarly, the high leakage current might also be verified through the Poole–Frankel and hopping conduction mechanism from the *I*-*V* curves of the electrical fitting diagram for the HRS state in Figure 8.

To analyze and investigate the reaction mechanism of the LRS state transitioning to the HRS state in BST thin film resistive RAM (RRAM) devices during the reset process, the switching conduction mechanism and initial electrical conduction formation model based on the decay reaction equation in these devices were defined and utilized [12]. The decay reaction equation addressing the decay rate for the electrochemical reaction equation is

(4) $$r=-1/m\;\left(-d\left[A\right]\right)/\mathrm{dt}=k{\left[A\right]}^{m}{\left[B\right]}^{n}$$

In this context, *r* represents the reaction time, *k* denotes the reaction constant, [*A*] is the reactant concentration, and *m*/*n* indicates the reaction level. To illustrate the decay reaction in the reset process of BST films RRAM devices, the reactant concentration and reaction time was converted into a ln([*A*]) − *t* function for a first-level reaction:

(5) $$\left[\left(n\times q\right)/V\right]=mole/V=\left[X\right]$$

**Figure 14:**
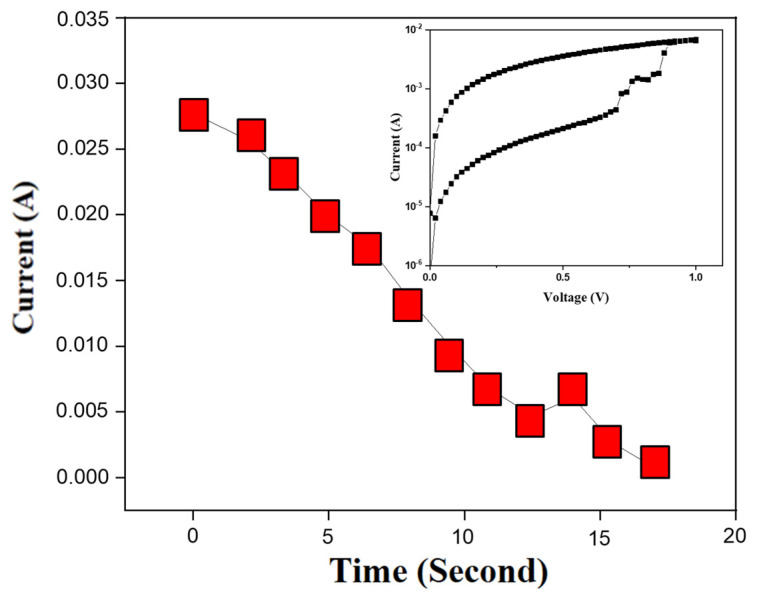
The electron charge of unit current (*A*) versus the reaction time (*S*) curves in the reset state of the BST film RRAM devices using a copper top electrode.

Equation (1) was used to determine the total charge quantity in *I*-*V* curves of BST film RRAM devices during the reset state for reactant concentration. The reactant concentration mole [X] is equal to n×q, where the reactant valence is *n*, and the charge of one mole electron is *q* (*q* = 96,500 C). The decay reaction considers the reaction time and concentration in BST film RRAM devices for the reset state. Figure 14 illustrates the reaction time versus current curves in the reset state from −1 to −0.5 V. The reset current was calculated and converted into charge versus reaction time curves. The equation for the total charge relationship for reaction time and operation current is below:

(6) $$Q\left(C\right)=I\left(A\right)\times \left(t\right)$$

The Q value was the integral of the current over time. To explore the first-level rate law for the electrochemical reaction in the reset state of BST film RRAM devices, the constant voltage sampling method was used to study the transition from LRS to HRS.

**Figure 15:**
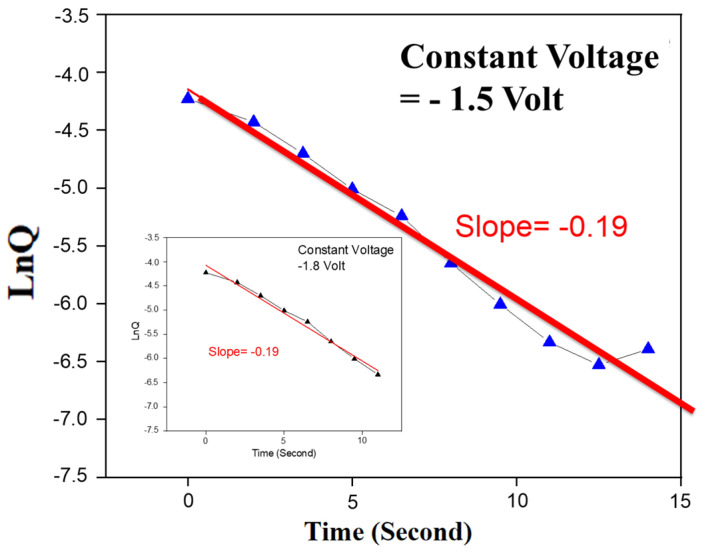
The ln*Q*-*T* curves of the BST thin film RRAM devices using a copper top electrode under −1.5V and −1.8 V constant voltage conditions.

The proportional reaction rate and reactant concentration of the first-level rate law in the electrochemical reactions were used to inspect properties involved in the reset process. In addition, the first-level decay reaction equation for oxide-based RRAM devices in the reset state was also determined and calculated. The derivation of the rate constant was successfully validated and obtained [12,39]. The relevant equations are described below. According to the equation showing the linear relationship between the natural logarithm of production concentration and reaction time,$\;K{\left[A\right]}^{1}$, where the reactant concentration is [*A*] and the reaction rate constant is *k*, the mole of the reactant is equal to *Q*/(*q*×*n*), where the quantity of the reaction is n, and the charge of one mole electron is q. Figure 15 illustrates the variation in total charge quantity (ln*Q*-*T*) for BST film RRAM devices during the reset state under a constant sampling voltage. The slope of the ln*Q*-*T* curves was examined in relation to the reaction rate. In accordance with the first-level law of the decay reaction, the slope of the ln*Q*-*T* curves in BST film RRAM devices at a constant sampling voltage of −1.5 V was calculated to be −0.19. Additionally, the slope of the ln*Q*-*T* curves in RRAM devices at −1.8 V was determined to be approximately −0.19. These experimental findings established the reaction rate constant in this study as −0.19. The constant sampling voltages showed a similar reaction rate across all RRAM devices. The first-level decay reaction equation for a BST thin film in the reset state was determined to be $r=0.19{\left[O^{2-}\right]}^{1}$. For Ni:SiO_2_ and vanadium film RRAM devices, slopes of 0.14 and 0.16 were also found for the first-level decay reaction constant [12,39].

**Figure 16:**
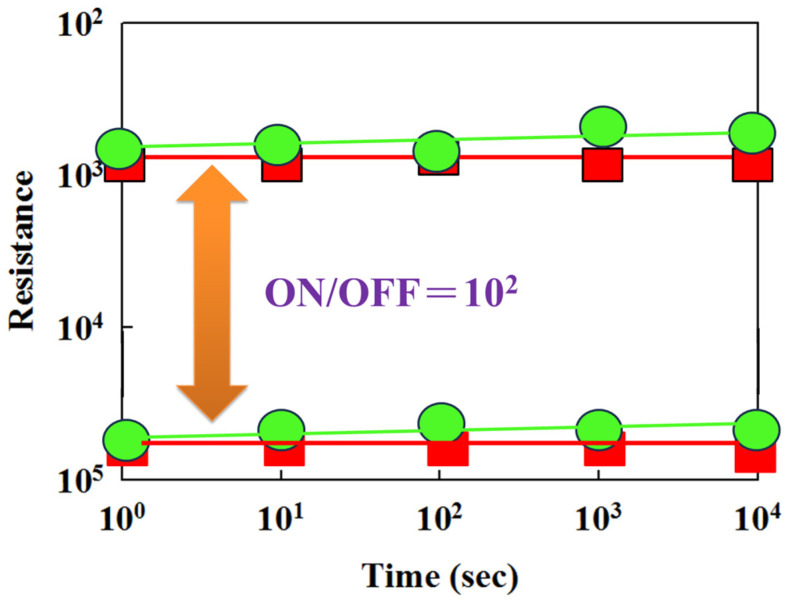
The resistance value versus time curves of the BST thin film RRAM devices (green: copper; red: aluminum).

**Figure 17:**
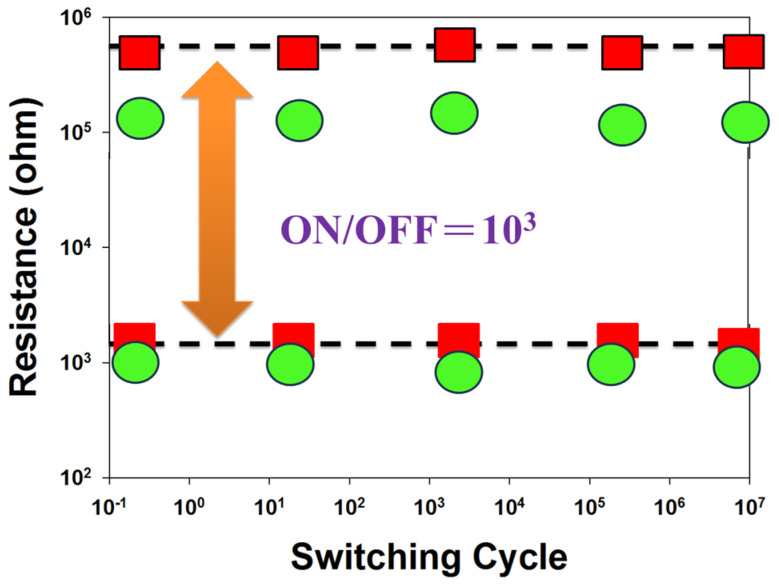
The resistance value versus switching cycle curves of the BST thin film RRAM devices (green: copper; red: aluminum).

Figure 16 shows the resistance value versus switching cycle curves of BST thin film RRAM devices for different materials: red represents Al and green represents Cu. It also indicates that the on/off ratio switching behavior cycling versus time curves remained stable for over 10^2^ seconds in the extrapolation calculation. Figure 17 illustrates the properties of the switching cycling versus resistance value curves, as measured using retention and endurance assessment methods. It displays the retention characteristics of BST thin film RRAM devices with various top-electrode materials (Al, Cu) to determine their reliability for use in non-volatile memory RRAM applications. As depicted in Figure 17, the on/off ratio switching resistance cycling versus testing time curves exhibit no significant variations in the BST thin film RRAM devices for over 10^3^ seconds, based on extrapolated calculations.

**Figure 18:**
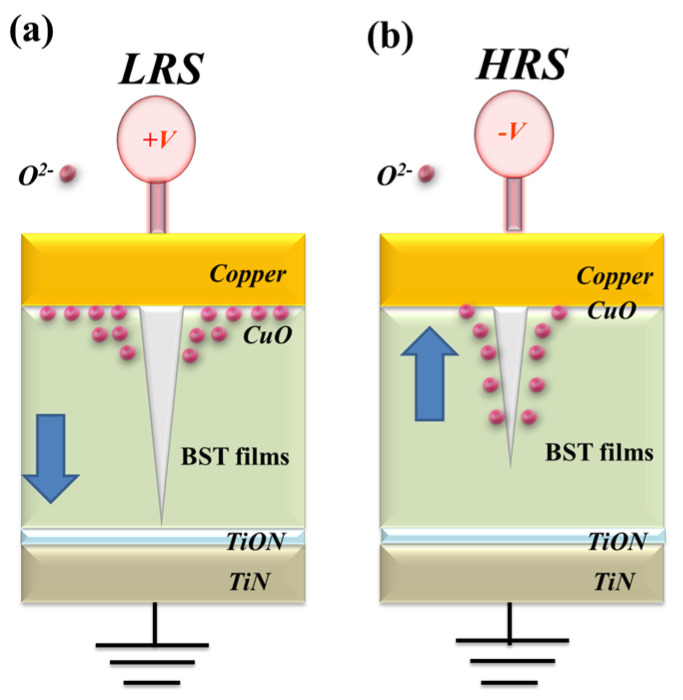
The conduction transfer mechanisms and metallic filament path model of the BST film RRAM devices for copper electrodes for the (**a**) LRS and (**b**) HRS states.

In Figure 18, oxygen vacancies are observed in the interface region between the copper top electrode and BST film of the RRAM device, accumulating progressively during LRS states. The physical conduction model causes a continuous oxidation reaction in the thin metallic filament path when high positive voltage is applied to the negative electrode. Thin metal filaments were influenced by oxygen atoms near the bottom electrode area. Figure 18 illustrates the electrical transfer mechanisms and the initial metallic filament path model of the film RRAM devices with a copper electrode in the set/reset state. In copper top-electrode RRAM devices, as opposed to aluminum top-electrode devices, the electrical conduction mechanism demonstrates ohmic conduction at low applied voltages. This phenomenon is attributed to defects, copper diffusion behavior, and high leakage currents near the interface of the copper top metal electrode and BST films within the RRAM device structure. Therefore, the Cu/BST/TiN (CBRAM) devices exhibited low on/off ratios, high leakage current, and poor ferroelectric properties for Al/BST/TiN (OxRAM) devices.

## 4. Conclusions

This study utilized the sol–gel method to spin-coat BST films on TiN/Si substrates. Subsequently, the BST films underwent post-treatment at different annealing temperatures to repair defects and rearrange the crystallization structure. Two types of top electrodes were fabricated, using sputtering to deposit copper and thermal evaporation to deposit aluminum as the top-electrode material, forming metal–insulator–metal structures. In addition, the ferroelectric and resistive switching characteristics of these two types of memory structures of CBRAM and OxRAM devices using copper and aluminum top-electrode materials were analyzed. The effects of different annealing temperatures on the BST films were investigated, and the memory characteristics under the two different top-electrode materials were compared.

From the resistive switching electrical measurements, it was known that the Cu/BST/TiN (CBRAM) structure might be used over 100 times at different annealing temperatures, with optimal performance at 500 °C, achieving approximately 10^2^ cycles, an on/off ratio of about 2, and an operating voltage of around 1 V. For the Al/BST/TiN (OxRAM) structure, only the device annealed at 500 °C could reach about 10^3^ cycles, with an on/off ratio of about 3.5 and an operating voltage of around 2 V. Based on the comprehensive analysis of resistive switching and ferroelectric properties, it was observed that the overall characteristics of CBRAM devices are superior to OxRAM devices. This was attributed to the formation of conductive filaments relying on metal electrode ions, which enhances the stability of switching between high- and low-resistance states compared to OxRAM devices. However, the on/off ratio decreases by 1.5 orders of magnitude, likely due to copper electrode diffusion reducing the switching ratio. Additionally, this device exhibited the ferroelectric properties, indicating that BST films not limited to applications in either resistive memory or ferroelectric memory, thus offering broader application value. Based on the excellent properties of BST ferroelectric films in RRAM devices, there are opportunities to apply them to CMOS integration devices and different ferroelectric memory material components in the future [40,41,42].

## Data Availability

No new data were created or analyzed in this study.
